# Major depressive disorder in post-secondary students attending foodbanks in France

**DOI:** 10.3389/fpubh.2023.1177617

**Published:** 2023-06-22

**Authors:** Lison Rambliere, Clémentine Leservoisier, Ysé Bedo, Melissa Macalli, Amandine Lebugle, Caroline Douay, Lorraine Guénée

**Affiliations:** ^1^Observatoire du Samusocial de Paris, Samusocial de Paris, Ivry-sur-seine, France; ^2^University of Bordeaux, Inserm, Bordeaux Population Health Research Center, U1219, CHU Bordeaux, Bordeaux, France

**Keywords:** mental health, precarity, student, foodbanks, depression, France

## Abstract

**Introduction:**

Poor mental health among youth is a major public health issue that has risen to the forefront since the COVID-19 crisis, especially among post-secondary students and precarious populations. The objectives of this work were to evaluate the rate of major depressive disorder (MDD) among precarious post-secondary students in the greater Paris region, to describe its risk factors, and to identify determinants for not seeking care.

**Methods:**

We conducted a multi-site, cross-sectional survey of post-secondary students attending a selection of 13 student foodbanks in the greater Paris region (France) between 30 November 2021 and 27 January 2022. This study had two complementary epidemiological and sociological components: a quantitative description of MDD through completion of a questionnaire performed through face-to-face or telephone interviews, and a qualitative assessment of the factors underlying MDD through in-depth follow-up interviews conducted among a sub-selection of students who participated in the first phase.

**Results:**

Among 456 students who participated in our survey, 35.7% presented with MDD. The risk of suffering from MDD was higher among women, students housed by third-parties, students reporting moderately to severely hungry and/or poor physical health. Students receiving material and/or social support were less likely to present with MDD. Among students who reported needing health care in the last year or since their arrival in France, 51.4% did not seek care.

**Conclusion:**

To address poor mental health among precarious students, policy action must jointly consider financial precarity, administrative barriers, housing, food security, physical health, and access to health services, especially mental health.

## 1. Introduction

Poor mental health among youth is a major public health issue that has come to the forefront globally since the onset of the COVID-19 pandemic (1, 2). Poor mental health among young adults, encourages risk-taking behaviors and is itself a risk factor for eating disorders, suicidal ideation and addiction (3–5). In France, suicide is the second leading cause of death after traffic accidents among 15- to 24-year-olds, accounting for 16.2% of all deaths (6).

Due to various reasons (e.g., financial difficulties, anxiety about the future), post-secondary students are a particularly vulnerable youth population, and challenges brought about by COVID-19 and public health measures enacted in response to the pandemic, such as social isolation due to distance learning, have had negative impacts on youth mental health (3, 7). For example, the share of post-secondary students in France experiencing major depressive disorders (MDD) was estimated at between 7% and 20% prior to the pandemic (8–11), and between 30% and 33% during the lockdown period (12).

Student populations are confronted with multiple forms of insecurity, including those related to finances, accommodation and administration, which can have negative health consequences (13). For instance, being unable to anticipate one’s budget on a regular basis necessitates rationing one’s expenses, and can lead to situations of precarity, feelings of vulnerability and a loss of control over one’s daily life. Moreover, poorer and more precarious populations suffer from a disproportionately high risk of many common mental disorders (14), and precarity is also known to be an important risk factor for not seeking health care, particularly among students and young adults (12, 15, 16). Although these insecurities are often transitional, with students achieving greater stability upon obtaining a more satisfactory social position after graduation, they can nonetheless last for months or years at a time and introduce significant mental health challenges in students’ daily lives (17).

Precarity can be defined as the absence of at least one security that allows people to benefit from their fundamental rights (18). Students requiring emergency food aid likely experience significant food insecurity, among potentially other forms of precarity (18). Such precarious populations experience significant economic and social difficulties and represent a particularly vulnerable segment of the student population (14). Since the beginning of the 2020–2021 academic year, non-profit organizations have been opening food distribution sites for students in France and have expressed concern about their high rates of attendance. However, in 2022 the French Court of Accounts highlighted the lack of reliable data on student precarity, mental health and health care seeking behavior, making it difficult for public authorities to make informed decisions to handle these overlapping crises (19). Yet gathering data on the most precarious segments of vulnerable populations is notoriously challenging.

The objectives of this study were to: (1) describe the socio-demographic characteristics of precarious post-secondary students attending foodbanks in the greater Paris region; (2) estimate the proportion of this population presenting with MDD; (3) identify risk factors associated with MDD; and (4) estimate the proportion of students not seeking health care, including mental health care.

## 2. Methods

### 2.1. Population-based survey

We conducted the EtuCris study (Étudiantes et étudiants en Crise), a multi-site, cross-sectional survey of post-secondary students attending a selection of 13 student foodbanks in in the greater Paris region (Île-de-France) between 30 November 2021 and 27 January 2022. By this date, all COVID-19 lockdowns and curfews had been lifted for approximately 6 months. Students had thus returned to in-person university classes after more than a year of distance learning. Characteristics of the included foodbanks are described in Supplementary material 1. Over the study period, a team of study investigators (trained in population-bases field studies) were positioned at foodbank exits and invited all individuals leaving the foodbank to participate in an interview. Students were presented with the option of participating either immediately in the form of a live interview, or later in the form of a follow-up telephone call at a time of their convenience.

Study investigators were present at each foodbank over multiple attendance slots in order to capture as many students as possible. To be eligible for inclusion, students had to be present at one of the 13 student food distribution sites on the survey day and enrolled in post-secondary studies in 2020–2021 and/or 2021–2022. Minors, parents of students, previously surveyed students, and students who did not speak English or French (rare cases) were excluded.

During interviews, study investigators completed questionnaires designed to collect data on students’ socio-demographics characteristics, educational status, housing, administrative situation (e.g., immigration status, access to social security), financial resources, use of social support systems, hunger as measured by the Household Hunger Scale (HHS), and their perceived physical and mental health (20). Answers were entered directly into a dedicated online data collection interface (Wepi®). The question of whether to enroll in a public or private university was raised because, in France, public universities cost around 300 euros per year, while tuition fees in private tertiary education institutions can amount to several thousand euros per year.

### 2.2. In-depth follow-up interviews

A second round of in-depth, follow-up interviews was conducted between 22 December 2021 and 13 April 2022 among a sub-selection of students who participated in our initial survey. Individuals included for participation were selected by consensus among the study investigators trained in sociology. Students were selected on a rolling basis over the course of the initial survey period, and prioritized students with contrasting profiles in terms of mental health status, nationality, administrative and residential barriers, impacts of the COVID-19 crisis, use of food aid, place of study, and food insecurity. Follow-up interviews were semi-structured and a variety of topics were covered, including details on the students’ use of food assistance, financial resources, family assistance, relationship to employment and health difficulties. Most follow-up interviews were recorded and all were subsequently transcribed. Interviewers also completed a field diary during follow-up interviews to note and share their observations.

### 2.3. Qualitative analyses

Qualitative analyses were conducted to describe the views expressed by the study population participating in our in-depth follow-up interviews. First, all information reported by students that was considered to be either directly or indirectly related to their mental health was grouped by theme (e.g., mental or physical health problems, difficulties in health care access). This work served as the basis for the development of a Directed Acrylic Diagram (DAG, Supplementary material 2) by a multidisciplinary team. DAGs have found application in the field of epidemiology as a tool to describe causal relationships between variables and to identify the variables that need to be included to control for confounding factors. Here, the DAG was used to select the variables to be included in the statistical model to identify the factors that are mechanistically involved in the risk of MDD. In addition, qualitative analyses have proven useful in the later stages of analyzing quantitative questionnaires (21). In this study, qualitative analyses were used to categorize open-ended responses, to identify any limitations in a priori categorization, to cross-validate responses given by the same respondents during both questionnaires and in-depth interviews, to aid in the interpretation of questionnaire responses, and to identify potential limitations in the questionnaire design.

### 2.4. Statistical analysis

Quantitative analyses were conducted to describe the study population participating in our population-based survey. The primary outcome considered was presentation with MDD, which was measured using standardized questions in our survey derived from the European Mini-International Neuropsychiatric Interview (MINI) (15). All students with complete data on mental health status were included in the analysis [21/496 (4.2%) students did not want to answer to at least one of the questions]. Descriptive statistical analyses were conducted. Proportions were compared using Chi2 tests. A value of *p* of less than 0.05 was considered significant. Major results are presented with 95% confidence intervals (95%CI).

Finally, to identify risk factors associated with MDD in the survey population, logistic regression models were constructed. Univariable models and a multivariable model considering all variables were conducted. The variables included were selected *a priori* according to the DAG resulting from our qualitative analysis and from data available in the literature. All variables with a direct causal effect on MDD were included in the final model [sex, nationality, age, housing and university type, resources (job, scholarship, financial difficulties), frequency of foodbank attendance, material and social support, administrative challenges, mode of questionnaire administration and other health issues (physical health and hunger)]. Missing data (<0.9% in only three co-variables) were replaced by multiple imputation using the mice package. A complete database was used to perform the analysis. A sensitivity analysis was performed with concordant results. Analyses were performed in R version 4.1.1.

Final results are presented as adjusted Odds Ratios (aOR) and 95%CI from the multivariate model, presented alongside conclusions derived from the qualitative analyses.

### 2.5. Ethics

The study was authorized by the legal department of Samusocial de Paris and was conducted in partnership with all participating foodbanks. Written, informed consent was obtained for all participants. Students were free to participate in the survey and it did not affect their food allocation, as interviews were proposed after students had already left the foodbank. Survey data were collected in a software accredited to store health data and were accessible only to a limited number of accredited individuals. Personal identifying data (names and phone numbers) were stored in an independent database. Recordings and transcriptions of in-depth interviews were available only to the study participants conducting the interviews, and corresponding field logs were pseudonymized and password protected. Names of students presented in the results were changed to preserve anonymity.

## 3. Results

### 3.1. Study population

**Figure 1:**
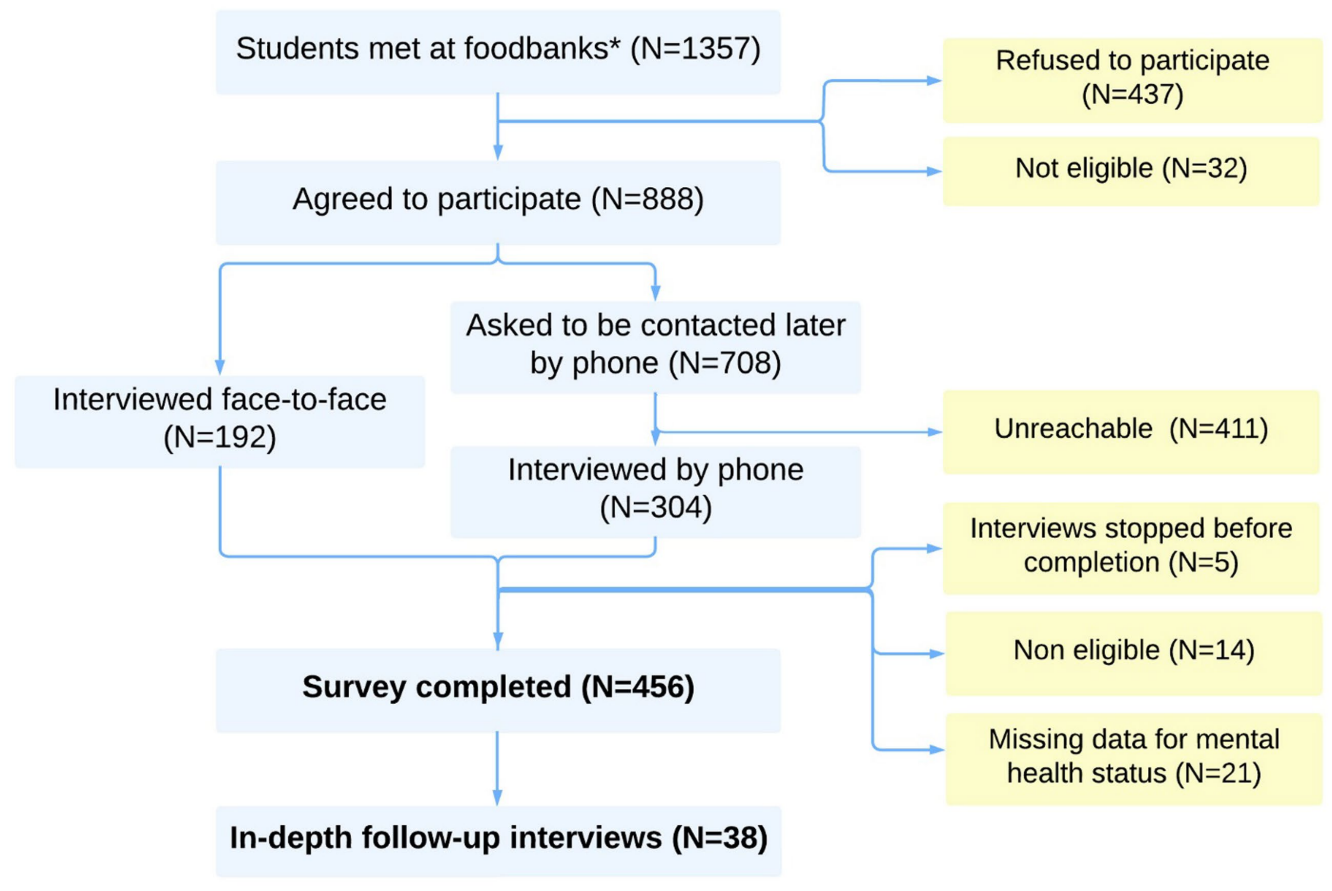
Study flow-chart (Etucris Study—2021). *Note that fewer than 1,357 unique individuals were invited to participate, as some individuals may have been approached multiple times by study investigators.

During the study period, 1,357 students were met at foodbanks, but some were potentially met multiple times (Figure 1). In total, 888 unique individuals agreed to participate in this study. Of these, 180 (20.3%) were directly interviewed face-to-face. Of the 708 who asked to be contacted later by telephone, 297 (41.9%) answered our call. Of the 477 interviews completed, 456 (91.9%) could be used in this analysis.

Among these 456 students, 60.1% were women, the median age was 24 years [interquartile range 18–26]. The vast majority were international students (80.3%), among whom 77.4% arrived in France for the new academic year (2021–2022, Table 1) and more than half (54.1%) were born in North Africa. Two thirds (67.8%) were enrolled in a public university. Student residence was the most reported type of housing (45.0%), of which two thirds (63.9%) were private residences. At the time of the questionnaire, 15.8% were hosted by a third-party, and only 1.5% of students reported living in a parent’s home.

**Table 1:** Characteristics of participating students (EtuCris study—2021).

		Mode of administration			Data missing
Variable	Overall *n* (%)	Telephonic *n* (%)	Face to face *n* (%)	*p*	
*n*	456	289	167		
Sex (male)	182 (39.9)	168 (58.1)	106 (63.5)	0.306	0
Nationality				0.056	0
French	90 (19.7)	48 (16.6)	42 (25.1)		
Foreign in France > 12 months^*^	160 (35.1)	101 (34.9)	59 (35.3)		
Foreign in France < 12 months^*^	206 (45.2)	140 (48.4)	66 (39.5)		
Age				0.777	0
<23 years	141 (30.9)	86 (29.8)	55 (32.9)		
23–25 years	182 (39.9)	117 (40.5)	65 (38.9)		
>25	133 (29.2)	86 (29.8)	47 (28.1)		
Housing type				0.439	0
Student residence	205 (45.0)	131 (45.3)	74 (44.3)		
Rental with a personal lease	154 (33.8)	103 (35.6)	51 (30.5)		
Hosted by a third-party	72 (15.8)	41 (14.2)	31 (18.6)		
Sublease	25 (5.5)	14 (4.8)	11 (6.6)		
Enrolled in public university (yes)	309 (67.8)	198 (68.5)	111 (66.5)	0.729	0
Degree being pursued				0.004	0
Bachelor’s degree	157 (34.4)	91 (31.5)	66 (39.5)		
Master’s degree	267 (58.6)	184 (63.7)	83 (49.7)		
Other	32 (7.0)	14 (4.8)	18 (10.8)		
Works alongside studies (yes)	164 (36.0)	108 (37.4)	56 (33.5)	0.471	4
Receives a scholarship (yes)	99 (21.7)	53 (18.3)	46 (27.5)	0.029	0
Major financial difficulty (yes)	314 (69.0)	201 (69.6)	115 (68.9)	0.962	1
Material support from relatives (yes)	285 (63.1)	183 (63.3)	102 (62.6)	0.955	4
Social support (yes)	357 (78.3)	219 (76.0)	123 (74.5)	0.808	0
Frequency at foodbank < 1/week	217 (47.6)	144 (49.8)	73 (43.7)	0.245	0
Poor physical health	105 (23.0)	50 (17.3)	55 (32.9)	<0.001	
Hunger index				0.224	3
Low	335 (74.0)	39 (13.6)	29 (17.5)		
Moderate	68 (15.0)	28 (9.8)	22 (13.3)		
Serious	50 (11.0)	220 (76.7)	115 (69.3)		
Health care renunciation^*^	166 (36.4)	93 (32.2)	73 (43.7)	0.099	0
Health insurance				0.404	0
Yes	322 (70.6)	200 (69.2)	122 (73.1)		
Waiting for permanent documents	116 (25.4)	79 (27.3)	37 (22.2)		
No	18 (3.9)	10 (3.5)	8 (4.8)		
Waiting for residence permit or visa	52 (11.4)	37 (12.8)	15 (9.0)	0.278	0
Waiting for financial assistance for housing	123 (27.0)	89 (30.8)	34 (20.4)	0.021	0
Waiting for a work permit	46 (10.1)	30 (10.4)	16 (9.6)	0.911	0
At least one administrative hurdle^**^	229 (50.2)	79 (48.5)	150 (51.2)	0.645	0

* At least once during the past year or upon arrival in France if this arrival took place less than a year ago.

** Defined as at least one of this event: waiting for residence permit or visa, waiting for permanent documents for health insurance, waiting for financial assistance for housing or waiting for a document to work.

Approximately one third of students (36.0%) were employed. Around 21.7% of students received a scholarship, respectively 56.7% of French students (mainly a French public scholarship) and 13.1% of foreign students (mainly scholarships from their country of origin). However, 69.0% reported major financial difficulties the month prior to the interview (unable to pay monthly expenses and/or pay rent). Around 63.1% received material support from relatives such as shared meals, and 78.3% reported having someone to rely on. Over the 30 days prior to their participation, 30.1% reported at least once having no food to eat and 26.3% said they had to sleep hungry because of a lack of food at home. While 52.4% reported going to the foodbank at least once a week, 26.0% had a moderate or serious hunger index.

During in-depth interviews, three principle profiles emerged from our survey: international students reporting recent arrival in France, international students arriving later in France who want more financial independence, and French students reporting disadvantaged backgrounds and relationship difficulties with their families. Moreover, students reported that student housing, especially public housing, was an important factor in saving money and maintaining social connection.

“I will be surrounded by students, it will be even better for me to have an entourage that allows me to develop, to meet people.” *Interview with Nesrine, Algerian, age 25, January 12, 2022*.

### 3.2. Mental health status

Self-rated physical health and mental health were rated as good to very good in 77.0% and 45.5% of students, respectively. Among all interviewed students, 5.3% reported attempting suicide at least once in their lives, this rate being 3.0% for international students and 14.4% for French students. The median age of the most recent suicide attempt was 17 years [interquartile range, 15–20].

In the last 30 days, more than half (52.0%) of the students reported feeling particularly sad, down or depressed, most of the time during the day and almost every day; and 42.5% felt they had lost interest in things they used to enjoy (Table 2). For 37.3% of students, their appetite had changed or they self-reported that they had unintentionally gained or lost weight, and 35.9% reported having problems sleeping almost every day. All these indicators were statistically higher for women compared to men. One in 10 students (10.4%) repeatedly had dark thoughts, thought they would be better off dead, or thought about hurting themselves.

**Table 2:** Mental health status among precarious student and by sex (EtuCris 2021–2022).

Variable	Overall *n* (%)	Man *n* (%)	Woman *n* (%)	*p*
*n*	456	182	274	
Felt particularly sad, blue, depressed, most of the time during the day almost every day	237 (52.0)	79 (43.4)	158 (57.7)	0.004
Feeling of having no taste for anything, of having lost interest in the things you used to enjoy	194 (42.5)	64 (35.2)	130 (47.4)	0.012
Appetite has changed/unintentionally gained or lost weight	170 (37.3)	51 (28.0)	119 (43.4)	0.001
Sleeping problems almost every night	163 (35.9)	53 (29.1)	110 (40.4)	0.018
Did things more slowly than usual/found it difficult to stay in place, almost every day	172 (37.7)	50 (27.5)	122 (44.5)	<0.001
Tired all the time, no energy	197 (43.2)	58 (31.9)	139 (50.7)	<0.001
Felt worthless or guilty almost every day	124 (27.2)	38 (20.9)	86 (31.4)	0.018
Have trouble concentrating or making decisions most days	191 (41.9)	61 (33.5)	130 (47.4)	0.004
Repeatedly had dark thoughts, thought you’d be better off dead or thought about hurting yourself	49 (10.7)	15 (8.2)	34 (12.4)	0.210
Major depressive disorder	163 (35.7)	49 (26.9)	114 (41.6)	0.002

Standardized questions derived from the European Mini-International Neuropsychiatric Interview (MINI).

Overall, 35.7% [95%CI 31.3–40.3] of students were classified as presenting with MDD according to MINI criteria, with a higher rate among women (41.6%, [35.7–47.7], *p* = 0.002) than men (26.9%, [20.6–34.0], Table 1). MDD was more frequently reported in face-to-face interviews (*n* = 180) than in telephone interviews (*n* = 297, 45.6% vs. 32.7%, *p* = 0.006), while the main socioeconomic characteristics did not differ statistically between these two groups (Table 1).

Half (50.0%) of students reported a deterioration of their mental health due to the COVID-19 crisis, or 70.5% among those presenting with MDD. During in-depth interviews, students were able to share at length how the COVID-19 crisis has impacted their health. For example, Moundir, a 26-year-old French student, reported that he had gained more than 20 kilograms during this period, which had triggered a relapse of his eating disorder (bulimia). Another 19-year-old French student, Irene, also concerned by weight gain and bulimic episodes, described the period of distance learning as the starting point of psychological difficulties. In good mental and physical health prior to the crisis, she reported currently being on antidepressants (prescribed by a physician) to calm her severe anxiety and eating disorders.

### 3.3. Risk factors

In the multivariable logistic regression model (Table 3), a higher risk of presenting with MDD was found for women (aOR = 1.89, CI95%[1.19–3.02]), students hosted by a third-party compared to those with a lease or sublease (2.80 [1.47–5.32]), those with moderate or severe hunger (1.72 [1.05–2.81]), and those reporting poor physical health (2.73 [1.65–4.54]). Those who received social support (0.60 [0.36–0.99]) were at lower risk of MDD.

**Table 3:** Risk factors of major depressive symptoms among precarious student (logistic regression, Etucris study—2021).

		Univariable analysis			Multivariable analysis		
Variable	*n*	OR	CI95%	*p*	aOR	CI95%	*p*
**Sex**							
Man	182	-	-		-	-	
Woman	274	1.93	1.29–2.92	0.001	1.89	1.19–3.02	0.007
**Nationality**							
International	366	-	-		-	-	
French	90	1.99	1.24–3.17	0.004	1.70	0.91–3.18	0.097
**Age**							
18–22 years	315	-	-		-	-	
>22 years	141	0.63	0.42–0.95	0.026	0.73	0.45–1.17	0.185
**Housing type**							
Rental with a lease or sublease	179	-	-		-	-	
Hosted by a third-party	72	2.31	1.31–4.08	0.004	2.80	1.47–5.35	0.002
Student residence	205	1.62	1.05–2.50	0.029	1.47	0.92–2.37	0.107
**Enrolled in public university**							
No	147	-	-		-	-	
Yes	309	1.07	0.71–1.62	0.747	1.02	0.65–1.62	0.926
**Work alongside study**							
No	292	-	-		-	-	
Yes	164	0.79	0.53–1.18	0.253	0.87	0.55–1.37	0.549
**Receives a scholarship**							
Yes	99	-	-		-	-	
No	357	0.73	0.47–1.16	0.184	1.01	0.57–1.82	0.984
**Major financial difficulty**							
No	316	-	-		-	-	
Yes	140	1.25	0.82–1.88	0.294	0.90	0.55–1.46	0.674
**Frequency at foodbank**							
At least 1 a week	239	-	-		-	-	
<1/week	217	0.75	0.51–1.10	0.139	0.88	0.57–1.34	0.547
**Material support from relatives**							
No	168	-	-		-	-	
Yes	288	0.59	0.40–0.88	0.009	0.65	0.42–1.02	0.060
**Social support**							
No	111	-	-		-	-	
Yes	345	0.57	0.37–0.88	0.010	0.60	0.36–0.99	0.045
**Hunger index**							
Low	337	-	-		-	-	
Moderate to serious	119	2.20	1.44–3.38	<0.001	1.72	1.05–2.81	0.029
At least one administrative blocking*							
Yes	227	-	-		-	-	
No	229	0.90	0.61–1.32	0.577	1.25	0.79–2.00	0.343
**Poor physical health**							
Yes	351	-	-		-	-	
No	105	3.76	2.40–5.96	<0.001	2.73	1.65–4.54	<0.001
**Mode of administration**							
Face-to-face interviews	167	-	-		-	-	
Telephone call	289	0.63	0.43–0.94	0.022	0.82	0.53–1.27	0.372

-, reference.

* Defined as at least one of this event: waiting for residence permit or visa, waiting for permanent documents for health insurance, waiting for financial assistance for housing or waiting for a document to work.

In in-depth interviews, Lina, an 18-year-old French student, reported that food insecurity causes her great stress. Her access to food depends solely on food aid and family support, which is sometimes unstable due to limited financial resources in her nine-person home. Salim, an Algerian student, is a good example of the link between mental health, physical health and hunger status as he suffers from MDD, a chronic illness and severe hunger. His complex administrative situation had affected both his ability to work, to seek reimbursement for treatment, and to find adequate housing in a place where he could find social support. As he is unable to work, he has been using his savings for daily expenditures, which pushes him to restrict his diet and creates a feeling of anxiety. In addition, he expresses difficulties in understanding the French health care system and therefore in seeking the care he needs. This situation has a direct impact on his mental and physical health.

In in-depth interviews, it also emerged that being hosted by a third-party is seen as an essential support for newly arrived foreigners, but that, in the long-term, these accommodations located further away from student areas can lead to a sense of isolation and greater difficulty integrating. Moreover, it can lead to a form of dependency on the host individual, sometimes resulting in complicated situations, such as nights on the street.

“I went to my aunt’s house, for a few months I stayed there. Afterwards, things did not go very well, there were problems, lots of problems […] so from one day to the next I found myself outside, all alone, abandoned and all by myself“, *Interview with Paul, Cameroonian, 24 years old, April 16, 2022*.

### 3.4. Mental health care renunciation

Overall, 70.8% of students reported needing health care in the past year or since their arrival in France (if less than 1 year). This rate was higher for those presenting with MDD (82.8% vs. 64.2%, *p* < 0.001).

One third (36.4%, IC95% [32.0%–41.0%]) of all students reported health care renunciation, or 51.4% [45.8%–57.0%] among those reporting health care needs. Among the latter, not seeking health care was more common among those with MDD (62.2% vs. 43.6%, *p* = 0.001). The main reasons reported for not seeking health care were financial reasons (51.8%), actual or perceived lack of medical coverage (16.8%), and difficulties knowing where to go (13.2%) or how to make an appointment (10.2%). In in-depth interviews, administrative challenges also emerged as an important factor. For instance, those who did not have a permanent social security number were not always aware of their legal entitlements to reimbursement of healthcare expenses, and having to advance health care costs was also a major deterrent to seeking health care.

Among all students, 12.7% did not seek mental health care in the past year (or since arrival in France), or 40.5% among those with MDD. The main reasons reported for not seeking mental health care were financial reasons (51.7%), difficulties knowing where to go (19.0%) or how to make an appointment (12.1%), and actual or perceived lack of medical coverage (8.6%).

## 4. Discussion

Students attending foodbanks in Île-de-France were mainly financially precarious, often foreign and recently arrived in France. More than a third (35.7%) were facing MDD at the time of the interview, with an increased risk for women, people hosted by a third party, those who declared themselves to be in poor physical health and those not benefiting from social or material support. The rate of foregoing care was also particularly high, with 4 out of 10 students with MDD reporting not seeking mental health care.

The rate of MDD in this population (35.7%) is above the rate previously estimated in the general student population in France before the COVID-19 crisis [from 7% to 20% (8–11)] and during the lockdown period [from 30% to 33% (2, 12)]. Mental disorders are distributed along a gradient of economic disadvantage in society (22) which is consistent with particularly high rates of MDD in our study population. The majority (69%) of this population faced major financial resource difficulties. This precarity plays an important role in MDD and has a direct impact on self-confidence and academic performance (notably caused by a decrease in work and learning productivity) (23). This contributes to social inequalities by limiting professional prospects of these students. Efforts to prevent student precarity therefore appear to be a major element in the prevention of MDD among youth.

Poor physical health and hunger were identified as risk factors for MDD and these three determinants emerged in in-depth interviews as being particularly interconnected. This has been identified previously in the literature (24). Poor mental health can lead to poor physical health outcomes, increasing the risk of communicable and non-communicable diseases, and both unintentional and intentional injuries (4). Conversely, physical health problems can increase the risk of mental disorders, complicating diagnosis and treatment (4). A systematic narrative review suggest a bidirectional association whereby food insecurity increases the risk of poor emotional health, and poor emotional health increases the risk of food insecurity (25). Similarly, food insecurity is associated with negative physical health outcomes (e.g., asthma, anemia, diabetes) and disruption of cognitive functioning, which can be explained by limitations in nutriment intake, poorer adherence to medical recommendations, and increased cortisol levels due to the stress caused by food insecurity (26). For these reasons, mental health, physical health, and food security are critical pillars for academic success (24, 27). For higher education to be an engine for reducing social inequality, immediate efforts must be made to prevent student food insecurity, and in turn to improve mental health and physical health outcomes. Joint screening across these three overlapping issues should be conducted among students by professionals and organizations involved in these areas.

Lack of social support and housing difficulties (particularly for those hosted by a third party) were identified as having a significant negative impact on student mental health. It has been shown previously that type of housing has a direct impact on fostering social connections, which is a crucial factor for mental health (28). For students in our study hosted by a third-party, most of whom are foreigners and recently arrived in France, this type of housing offers opportunities to be welcomed upon their arrival and to save money. However, students also reported that this type of accommodation can become a major source of stress over time, due to insecurity regarding its stability and sustainability, and potential dependence upon their host. In addition, third-party accommodation is often found far from the student area, which can create feelings of isolation and difficulty integrating. Robust social support mitigates the negative effects of stress by improving self-esteem and coping skills, and by providing problem-solving strategies to better manage stress and, by consequence, risk of depression (29). Social support, self-esteem and self-rated health are closely correlated, with one predicting the other (30, 31). A major lever for MDD prevention is optimizing the student environment, living conditions and the fostering of adequate social supports for students.

Finally, MDD risk was twice as high in women compared to men, which has been reported previously in many studies (2, 10, 11, 32). This difference first appears around the age of 13, reaching a rate twice as high in girls as in boys by the end of adolescence (33). Several hypotheses have been put forward to explain this difference, such as the fact that girls and women are more exposed to gender inequality, to stress, are more reactive to relational disturbances (e.g., family conflicts, separation), or are more exposed to sleep disorders (9, 34).

The proportion of students not seeking care was high (51%). Finances, difficulties understanding and finding one’s way through the healthcare system, and administrative barriers were the most commonly reported reasons. This differs substantially from the general French student population, among whom the primary reasons for not seeking care are wanting to solve the problem on one’s own (e.g., waiting for health to improve, self-care), followed by other structural barriers [e.g., finances, lack of time; (16, 32)]. Students represent a young population who may face challenges entering into independent contact with the health care system and understanding its administration (3). Health literacy, particularly mental health literacy, is a key factor facilitating early detection of psychological problems and timely access to care (35). Further research is needed to evaluate health literacy levels among students, and in particular among precarious students such as those visiting foodbanks, most of whom are from foreign countries. Helping students to identify and access available mental health services seems essential to improve mental health care outcomes. Digital tools, outreach initiatives or playful educational activities implemented in student areas have proven effective for mental health prevention among students (36, 37). These programs may be particularly impactful if focusing on students most likely to suffer from MDD: precarious students, women, and those with housing difficulties or lacking social support.

The proportion of students not seeking care was even higher among those presenting with MDD (62.2%). Finances are a major barrier to seeking mental health care in general, as many mental health resources such as psychologist appointments are in most cases not reimbursed by French social security (3). High costs are a particularly important reason for not seeking care among precarious students, who must balance competing expenses, including rent, school fees, food, leisure and other health needs (38). Stigma is also a clear deterrent to seeking care for mental health. Certain groups appear to be particularly vulnerable to the influence of stigma, including younger people (who more often report mental health consultation as “not normal”) and men (who more often report “difficulty talking to professionals”) (39). Future interventions should incorporate broad approaches to limiting financial barriers and improving understanding of and access to the health care system, while also including stigma reduction strategies.

## 5. Strengths and limitations

The combination of epidemiological and sociological methods is a major strength of this study, allowing for both the identification and quantification of the mental health problems encountered by this student population. Thematic analysis of in-depth student interviews allowed us to identify the key factors underlying students’ self-reported mental health, to conceptualize the relationships between these factors in the form of a DAG, and in turn to include the most relevant factors as explanatory variables in our logistic regression model. Further, in-depth interviews allowed students to express themselves above and beyond the constraints imposed by a standard quantitative questionnaire, and allowed us to report concrete examples of students’ lived experiences, for instance strong overlap between students’ hunger, poor mental health and poor physical health. The objective of this study was not to conduct a thorough qualitative analysis of the physical and mental health issues experienced by these students. Nonetheless, our findings suggest that complementary sociological investigations could be highly valuable to better understand associations between precarity and mental health in this population.

One limit of our study is that participation was voluntary among students leaving foodbanks, and the sample may therefore not represent all impoverished or precarious students. For example, socially isolated students might not be aware of foodbank services, while students in more favorable circumstances who nonetheless use foodbanks may have refused to participate in our study about precarious students if they did not feel legitimate or identify as being one. However, our study design nonetheless allowed us to include a segment of the student population that is particularly difficult to capture in most general population surveys. As some students may have been approached several times, it was not possible to estimate the study inclusion ratio among students who attended foodbank during the study period.

In our study, we identified that the rate of MDD in our initial population survey varied across students responding via face-to-face interviews vs. those responding by telephone call, despite having similar demographic characteristics. Face-to-face interviews may have resulted in stronger bonds of trust between students and study investigators or a stronger willingness to testify, and hence more reliable responses to our questions. Further, students responding by phone may have been responding from the comfort of their own home and at a time of better convenience, resulting in a better perception of mental health at the time of the interview.

Finally, this cross-sectional study took place in part during the exam period, which may have resulted in some students responding to our survey during a particularly stressful period, as reported by some students during in-depth interviews. Future longitudinal studies including questions about exams and other time-variant stressors may allow for better description of MDD evolution over time.

## 6. Conclusion

This epidemiological and sociological study reports high rates of MDD in students attending foodbanks in the greater Paris region, and highlights the urgent need to address poor mental health among vulnerable youth. Approaches to address poor mental health will need to jointly consider financial precarity, administrative barriers, housing, food security and physical health, and ensure better access to mental health care.

## Data availability statement

The data supporting the findings of this study are available on request from the corresponding author, LR. The data is not publicly available due to ethical restrictions.

## Author contributions

LR was implicated in the creation of the questionnaire, the data management, conducted formal analysis of the data, interpreted the data, created the figures, and wrote the original draft. CL created the questionnaire, collected the data (interviews and in-depth interviews), and was implicated in the writing of the original draft. YB collected the data (interviews and in-depth interviews) and conducted the analysis of the in-depth interviews data. MM contributed to critical revision for important intellectual content on the data analysis and the manuscript. AL was implicated in the creation of the questionnaire and collected the data (interviews) and acquired funding for the study. CD conceived and designed the study and acquired funding for the study. LG conceived and designed the study, acquired funding for the study, collected the data (interviews and in-depth interviews), and conducted the analysis of the in-depth interviews data. All authors contributed to the article and approved the submitted version.

## Funding

This project was funded by the City of Paris, the Maison des Initiatives Étudiantes (MIE), the Regional Health Agency of Île-de-France, the Carasso Foundation, the Caisse nationale des allocations familiales (Caf), and Fondation de France (number 00109110).

## Conflict of interest

The authors declare that the research was conducted in the absence of any commercial or financial relationships that could be construed as a potential conflict of interest.

## Publisher’s note

All claims expressed in this article are solely those of the authors and do not necessarily represent those of their affiliated organizations, or those of the publisher, the editors and the reviewers. Any product that may be evaluated in this article, or claim that may be made by its manufacturer, is not guaranteed or endorsed by the publisher.

## Abbreviations

MDD: Major depressive disorder

## References

[1] Chêne G. Comment évolue la santé mentale des Français pendant l’épidémie de COVID-19? Résultats de la vague 34 de l’enquête CoviPrev (9-16 mai 2022), vol. 34. France: Santé publique France (2022). 9e p.

[2] Arsandaux J Montagni I Macalli M Texier N Pouriel M Germain R. Mental health condition of college students compared to non-students during COVID-19 lockdown: the CONFINS study. BMJ Open. (2021) 11:e053231. doi: 10.1136/bmjopen-2021-053231, PMID: 34413111 PMC8380475

[3] Egsdal M Montagni I Tournier M Tzourio C. Les services en santé mentale à disposition des étudiants inscrits dans l’enseignement supérieur: le cas de l’université de Bordeaux. Revue française des affaires sociales. (2016) 1:105–22. doi: 10.3917/rfas.162.0105

[4] Prince M Patel V Saxena S Maj M Maselko J Phillips MR. No health without mental health. Lancet. (2007) 370:859–77. doi: 10.1016/S0140-6736(07)61238-0 17804063

[5] Thapar A Eyre O Patel V Brent D. Depression in young people. Lancet. (2022) 400:617–31. doi: 10.1016/S0140-6736(22)01012-1 35940184

[6] CépiDc-Inserm. Données épidémiologiques sur les décès par suicide. France: Observatoire national du suicide (2017).

[7] Macalli M Texier N Schück S Côté SM Tzourio C. A repeated cross-sectional analysis assessing mental health conditions of adults as per student status during key periods of the COVID-19 epidemic in France. Sci Rep. (2021) 11:21455. doi: 10.1038/s41598-021-00471-8, PMID: 34753945 PMC8578661

[8] Tran A Tran L Geghre N Darmon D Rampal M Brandone D. Health assessment of French university students and risk factors associated with mental health disorders. PLoS One. (2017) 12:e0188187. doi: 10.1371/journal.pone.0188187, PMID: 29176864 PMC5703533

[9] Montagni I Qchiqach S Pereira E Tully PJ Tzourio C. Sex-specific associations between sleep and mental health in university students: a large cross-sectional study. J Am Coll Heal. (2020) 68:278–85. doi: 10.1080/07448481.2018.1546183, PMID: 30615574

[10] Léon C Chan Chee C du Roscoät E. La dépression en France chez les 18-75 ans: résultats du baromètre santé 2017. Bull Epidemiol Hebd. (2018) 33:637–44.

[11] Macalli M Côté S Tzourio C. Perceived parental support in childhood and adolescence as a tool for mental health screening in students: a longitudinal study in the i-share cohort. J Affect Disord. (2020) 266:512–9. doi: 10.1016/j.jad.2020.02.009, PMID: 32056920

[12] Chêne G. Comment évolue la santé mentale des Français pendant l’épidémie de COVID-19? Résultats de la vague 33 de l’enquête CoviPrev (8–15 avril 2022). France: Santé publique France (2022).

[13] Vial B. Comprendre la surexposition des jeunes aux difficultés administratives: une analyse critique des politiques publiques de jeunesse. France: Defenseur des droits. (2019).

[14] Allen J Balfour R Bell R Marmot M. Social determinants of mental health. Int Rev Psychiatry. (2014) 26:392–407. doi: 10.3109/09540261.2014.928270 25137105

[15] Wathelet M Duhem S Vaiva G Baubet T Habran E Veerapa E. Factors associated with mental health disorders among university students in France confined during the COVID-19 pandemic. JAMA Netw Open. (2020) 3:e2025591. doi: 10.1001/jamanetworkopen.2020.25591, PMID: 33095252 PMC7584927

[16] Baggio S Iglesias K Fernex A. Healthcare renunciation among young adults in French higher education: a population-based study. Prev Med. (2017) 99:37–42. doi: 10.1016/j.ypmed.2017.02.002, PMID: 28189805

[17] Sèze B. Précarité étudiante: vers l’autonomie sociale des jeunes? Études. (2021) 4280:35–48. doi: 10.3917/etu.4280.0035

[18] Bureau du CESE. 1987–2017: Poursuivre résolument la lutte contre la grande pauvreté | Le Conseil économique social et environnemental. (2023). Available at: https://www.lecese.fr/travaux-publies/1987-2017-poursuivre-resolument-la-lutte-contre-la-grande-pauvrete (Accessed 14 April 2023).

[19] Cour des comptes. Soutien de l’Etat à la vie étudiante. Rapport annuel (2022)101–134.

[20] Household Hunger Scale (HHS). Indicator definition and measurement guide | food and nutrition technical assistance III project (FANTA). (2023). Available at: https://www.fantaproject.org/monitoring-and-evaluation/household-hunger-scale-hhs (Accessed 20 January 2023).

[21] Les apports réciproques des méthodes quantitatives et qualitatives. Documents de travail—Ined éditions—Ined—Institut national d’études démographiques. Available at: https://www.ined.fr/fr/publications/editions/document-travail/les-apports-reciproques-des-methodes-quantitatives-et-qualitatives/ (Accessed 20 April 2023).

[22] World Health Organization. Social determinants of mental health. Switzerland: World Health Organization (2014).

[23] Eisenberg D Golberstein E Hunt JB. Mental health and academic success in college. B E J Econom Anal Policy. (2009) 10:45. doi: 10.2202/1935-1682.2191

[24] Farahbakhsh J Hanbazaza M Ball GDC Farmer AP Maximova K Willows ND. Food insecure student clients of a university-based food bank have compromised health, dietary intake and academic quality. Nutr Diet. (2017) 74:67–73. doi: 10.1111/1747-0080.12307, PMID: 28731560

[25] Bruening M Dinour LM Chavez JBR. Food insecurity and emotional health in the USA: a systematic narrative review of longitudinal research. Public Health Nutr. (2017) 20:3200–8. doi: 10.1017/S1368980017002221, PMID: 28903785 PMC10261670

[26] Gundersen C Ziliak JP. Food insecurity and health outcomes. Health Aff. (2015) 34:1830–9. doi: 10.1377/hlthaff.2015.0645 26526240

[27] Raskind IG Haardörfer R Berg CJ. Food insecurity, psychosocial health and academic performance among college and university students in Georgia, USA. Public Health Nutr. (2019) 22:476–85. doi: 10.1017/S1368980018003439, PMID: 30724722 PMC6366643

[28] Singh A Daniel L Baker E Bentley R. Housing disadvantage and poor mental health: a systematic review. Am J Prev Med. (2019) 57:262–72. doi: 10.1016/j.amepre.2019.03.018, PMID: 31326010

[29] Lee C Dickson DA Conley CS Holmbeck GN. A closer look at self-esteem, perceived social support, and coping strategy: a prospective study of depressive symptomatology across the transition to college. J Soc Clin Psychol. (2014) 33:560–85. doi: 10.1521/jscp.2014.33.6.560

[30] Wang X Cai L Qian J Peng J. Social support moderates stress effects on depression. Int J Ment Heal Syst. (2014) 8:41. doi: 10.1186/1752-4458-8-41, PMID: 25422673 PMC4242489

[31] Arsandaux J Michel G Tournier M Tzourio C Galéra C. Is self-esteem associated with self-rated health among French college students? A longitudinal epidemiological study: the i-share cohort. BMJ Open. (2019) 9:e024500. doi: 10.1136/bmjopen-2018-024500, PMID: 31167858 PMC6561426

[32] Tavolacci M-P Déchelotte P Ladner J. Eating disorders among college students in France: characteristics, help-and care-seeking. Int J Environ Res Public Health. (2020) 17:914. doi: 10.3390/ijerph17165914, PMID: 32824038 PMC7460404

[33] Girgus JS Yang K. Gender and depression. Curr Opin Psychol. (2015) 4:53–60. doi: 10.1016/j.copsyc.2015.01.019

[34] Pacheco JPG Silveira JB Ferreira RPC Lo K Schineider JR Giacomin HTA. Gender inequality and depression among medical students: a global meta-regression analysis. J Psychiatr Res. (2019) 111:36–43. doi: 10.1016/j.jpsychires.2019.01.013, PMID: 30665010

[35] Montagni I González Caballero JL. Validation of the mental health literacy scale in french university students. Behav Sci. (2022) 12:259. doi: 10.3390/bs12080259, PMID: 36004830 PMC9404754

[36] Montagni I Tzourio C Cousin T Sagara JA Bada-Alonzi J Horgan A. Mental health-related digital use by university students: a systematic review. Telemed J E Health. (2020) 26:131–46. doi: 10.1089/tmj.2018.0316, PMID: 30888256

[37] Montagni I Capelle A Chalifour C Langlois E. Rechercher et s’approprier l’information en santé mentale sur Internet: une étude qualitative auprès d’étudiants. rfsic. (2018) 4:39–50. doi: 10.4000/rfsic.5097

[38] Castry M Wittwer J Montagni I Tzourio C. Les déterminants du renoncement aux soins pour raisons financières des étudiants—une analyse à partir de l’étude i-Share|Cairn.info. Rev Econ Polit. (2019) 129:467–88. doi: 10.3917/redp.294.0467

[39] Clement S Schauman O Graham T Maggioni F Evans-Lacko S Bezborodovs N. What is the impact of mental health-related stigma on help-seeking? A systematic review of quantitative and qualitative studies. Psychol Med. (2015) 45:11–27. doi: 10.1017/S0033291714000129 24569086

