# Supplementary material for: Major depressive disorder in post-secondary students attending foodbanks in France
Source: Front Public Health. 2023 Jun 22;11:1177617. doi: 10.3389/fpubh.2023.1177617 (PMC10325617; doi:10.3389/fpubh.2023.1177617)

**Supplementary materials**

Supplementary 1: Short description of the student food distribution sites investigated.

The survey was conducted at 13 student food distribution sites (“foodbanks”) run by a total of six non-profit organizations: Co’P1 (n=2 sites), Linkee (n=5), Restos du Coeur (n=3), Secours Populaire (n=1), Agoraé (1), and On Remplit le Frigo (n=1). Each organization had its own mode of operation and student registration. For Co'P1, students register to receive food baskets and are invited to visit once every two weeks. For Linkee, students can access all sites after registration. For Restos du Coeur, students register, then provide additional documents to allow estimation of their living expenses and determine their food allocation. For Secours Populaire, could reserve slots in the solidarity grocery stores (but other publics were also welcomed), and also operates on the basis of students’ estimated living expenses, as determined during an interview with each student. For Agoraé, a solidarity grocery store accessible by application, students could buy products at 10% of the market price. Finally, On Remplit le Frigo is an itinerant organization which proposes food distribution in student areas, especially on campuses.

*Supplementary 2: Directed acyclic diagram (DAG)*


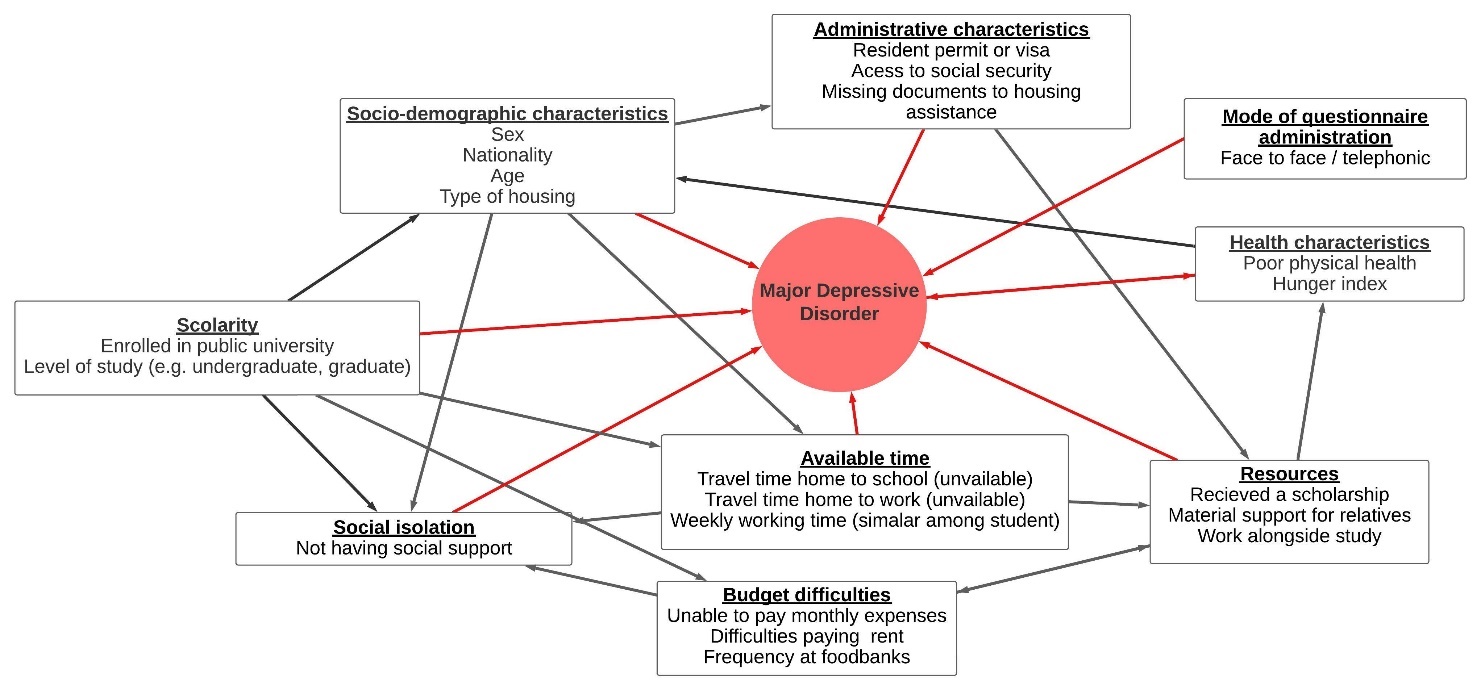

Supplement: Supplementary file 1 [file Data_Sheet_1.docx]
